# Canine epilepsy/seizure occurrence in primary care and referral populations: a look into the epidemiology across countries

**DOI:** 10.3389/fvets.2024.1455468

**Published:** 2024-10-01

**Authors:** Meaghan E. Bride, Francesca Samarani, Lauren E. Grant, Fiona M. K. James

**Affiliations:** ^1^Department of Clinical Studies, Ontario Veterinary College, University of Guelph, Guelph, ON, Canada; ^2^Department of Population Medicine, Ontario Veterinary College, University of Guelph, Guelph, ON, Canada

**Keywords:** canine epilepsy, prevalence, epidemiology, seizures, primary veterinary care, international, first opinion

## Abstract

Epilepsy is a common neurological condition in dogs. Analysis of primary care populations across countries can provide a more complete understanding of the epidemiology of this condition and provide context for spectrum of care discussions. This narrative literature review was aimed at understanding canine epilepsy/seizure prevalence in primary care populations, and changes in occurrence across geography, culture, and socioeconomic status. There are few studies to give insight into the true general population of epileptic canines and there is inconsistency in the literature regarding the standards applied for epilepsy diagnosis across primary care and referral practices. Therefore, the future focus should be on more epidemiological research in primary care and mixed populations, more veterinary education to standardize use of medical guidelines in primary care settings, and increased awareness of the benefits of having pet insurance to mitigate the potentially substantial cost of care for dogs with epilepsy.

## 1 Introduction

Epilepsy is a common neurological condition in dogs (1). It is defined as a disease that predisposes the brain to generate epileptic seizures (2). Seizures are not always epileptic, and are defined as any sudden, short, and transient event (2). The process of diagnosing, treating, and managing canine epilepsy is difficult as all observations and clinical decisions come from veterinarians and owners. As a result, many seizures go unrecognized or misclassified (3–5). Canine epilepsy affects a dog’s quality of life and causes immense stress on the caregiver (6). Caregivers report lifestyle restrictions out of fear of seizures, or even spontaneous death, occurring in their absence (6).

The International Veterinary Epilepsy Task Force (IVETF) defined three epilepsy types: idiopathic epilepsy (IE), structural epilepsy (StE), and epilepsy of unknown cause (EUC). IE is without a structural cause, where no cerebral pathology is confirmed or suspected, and seizure onset occurs between 6 months and 6 years of age (2, 7). There are three sub-classes: proven genetic background, suspected genetic background, and unknown cause (2). StE is caused by an identified cerebral pathology, while EUC has no known cause or classification, no indication of StE, and onset before 6 months and after 6 years old (2, 7). In one study, canine epilepsy of unknown origin (EUO) was previously defined as dogs experiencing more than two seizures for over a year, in the absence of comorbid conditions or with four or more antiseizure medications solely for seizure control (1).

The IVETF consensus proposals have standardized diagnostic protocols for IE, which are categorized through a tiered system providing a level of confidence in the diagnosis: Tier I involves at least two unprovoked seizures, with a diagnostic workup of blood tests, urinalysis, and unremarkable inter-ictal physical and neurological examinations. Tier II includes, in addition to Tier I, unremarkable fasting and post-prandial bile acid analyses, magnetic resonance imaging (MRI) of the brain, and cerebrospinal fluid (CSF) analysis. Tier III adds electroencephalography (EEG) to confirm seizure activity (7).

Prevalence measures the total number of dogs with epilepsy, including new and existing cases, within a defined population and time-period, reflecting both epilepsy risk and duration. Incidence counts the total number of dogs that newly develop epilepsy within a defined population and time-period, reflecting epilepsy risk. For this reason, measuring canine epilepsy incidence is preferred for identification and characterization of risk factors; however, almost all studies to date have measured prevalence (8–10). Further, most studies have analyzed referral canine populations (8–10). Referral populations include dogs that are affected by a more complicated or severe form of epilepsy, who have been referred to specialized care settings by a primary care veterinarian (11). These populations are often easier to access for research compared to primary care populations (11, 12) but are not representative of the general population of epileptic dogs, limiting inferences (13). For a more accurate understanding of the epidemiology of canine epilepsy, well-defined, representative canine populations should be studied. Primary care populations involve animals under routine veterinary care, where a general practice veterinarian handles their overall health. Analyzing primary care rather than referral populations provides a less biased estimate of the true prevalence of canine epilepsy within the general dog population. However, even primary care populations exclude those who are unable or opt not to access veterinary care.

Prevalence estimates of canine epilepsy globally enable preliminary identification of socio-demographic characteristics, such as geographic location, culture, or socioeconomic status. These characteristics influence risk of epilepsy in human populations (14–18), but their impact on canine populations is unknown. This narrative literature review aimed to describe canine epilepsy/seizure prevalence in primary care populations, and any differences in disease burden by geography, culture, and socioeconomic status. This review further provides insight into the state of canine epilepsy protocols across countries and how IVETF consensus proposals are used across primary care and referral establishments.

## 2 Materials and methods—search strategy

An electronic literature search performed in January and February 2023 used four academic databases—PubMed, CAB Direct, ProQuest, and Omni at the University of Guelph. Gray literature was searched using Google Scholar. Search terms used in the databases were: (canine OR dog) AND (epilepsy OR seizures OR seizure) AND (occurrence OR prevalence OR epidemiology OR incidence) AND [(primary OR first opinion) AND (veterinary OR veterinary medicine OR population OR health care OR care)]. Additional papers were found by hand-searching reference lists of relevant articles. Peer-reviewed articles regarding the prevalence of canine epilepsy and/or seizure occurrence across countries were identified without date restrictions. Articles must have discussed prevalence of canine epilepsy and/or seizures and identified whether a primary care or referral population was studied. Articles excluded were in a non-English language, reviews, clinical trials, studies discussing singular breeds, studies that only examined a subset of epileptic dogs, and studies that did not describe occurrence of canine epilepsy and/or seizures. These exclusion criteria avoided breed predispositions, limited patient populations, articles that did not provide testable data, and articles where epidemiology/prevalence of disorder was not one of their primary aims.

## 3 Results

**Figure 1:**
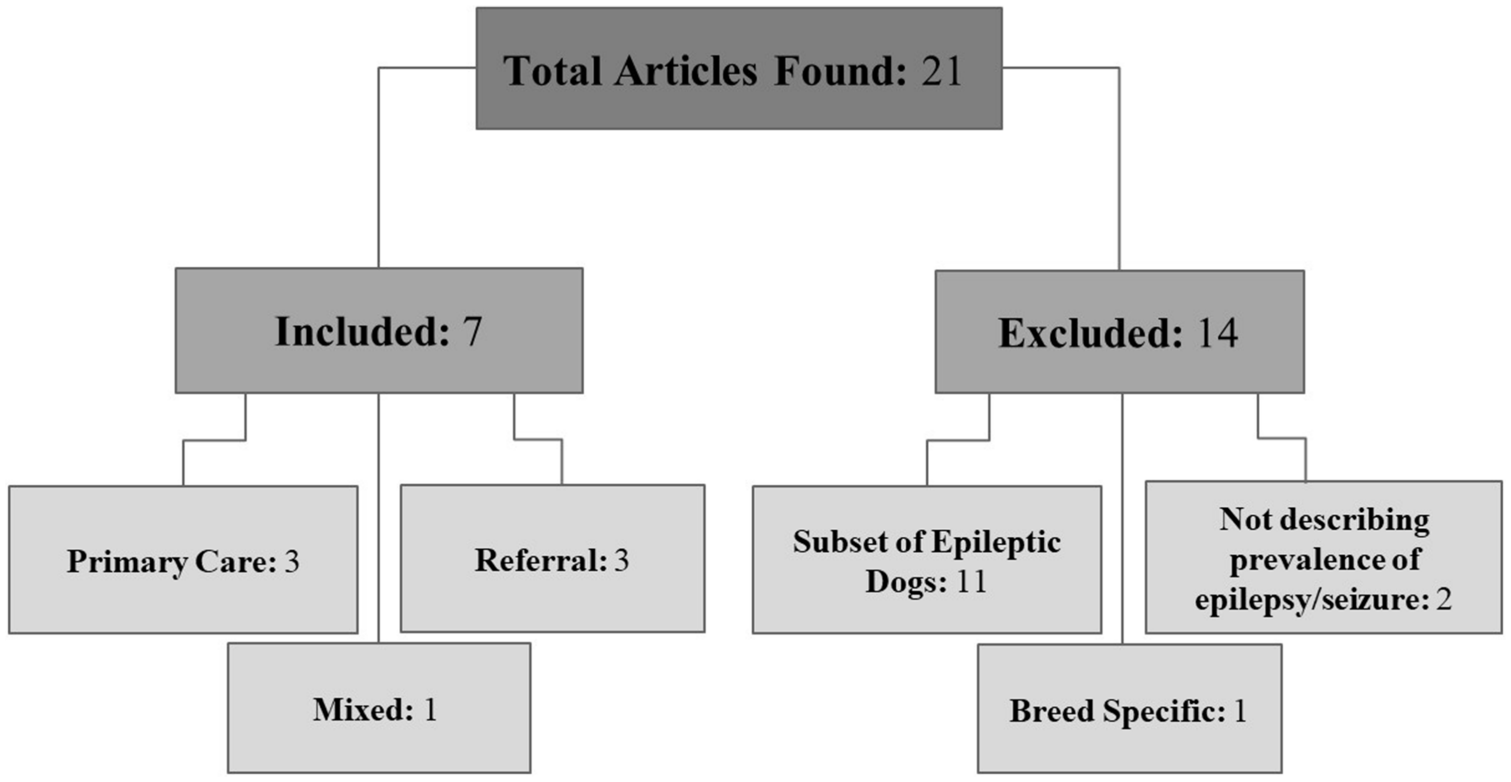
Article organization. Diagram of articles included and excluded from the review.

Out of 21 articles found, only seven fulfilled the inclusion criteria: three primary care, three referral, and one mixed population type (Figure 1). The term “mixed” was applied to a population of insured animals which included dogs in both primary care and referral practices.

### 3.1 Geographic distribution

All primary care articles came from the United Kingdom, referral papers came from the United States, Germany, and Japan, and the sole mixed population article came from Sweden.

All primary care articles used Vet Compass, an online database that collates first opinion patient data from hundreds of United Kingdom veterinary practices, for their retrospective studies (1, 11, 12). Across the primary care papers, Kearsley-Fleet et al. (1) found the 2-year prevalence of EUO to be 0.62% (539/87,317), Erlen et al. (11) found the 1-year prevalence for dogs having at least one seizure to be 0.82% (3,731/455,553) and Erlen et al. (12) found the 1-year seizure incidence risk to be 0.62% (2,834/455,553).

All referral studies used veterinary records from referral clinics for their retrospective studies. Across the referral papers, the epileptic seizure prevalence of the German dog population over a 6-year period was 2.6% (394/15,449) (8); the epilepsy prevalence among the Japanese dog population over a 10-year period was 1.9% (0.9% IE and 0.4% StE) (358/19,193) (9) while the IE prevalence among the American dog population was 1.04% (937/90,004) over a 15-year period (10). The mixed population paper retrospectively analyzed life and veterinary care insurance claims from Sweden. Heske et al. (4) found the 11-year epilepsy prevalence of the Swedish dog population to be 0.75% (5,071/665,249). Additionally, they identified incidence and mortality rates to be 18 cases per 10,000 Dog Years at Risk (DYAR) and 11 deaths per 10,000 DYAR, respectively (4).

### 3.2 Epilepsy diagnosis

The standard for epilepsy diagnosis differed across primary care and referral populations. For the estimated 70% of United Kingdom dogs that were under primary care, receiving a IVETF Tier I or higher diagnostic effort was not guaranteed especially for incident cases, i.e., dogs with no previous history of seizures (11, 12). Erlen et al. (12) found only 8.6% (245/2,834) of incident cases were diagnosed with epilepsy. Out of those diagnosed, 33.1% (81/245) received a diagnostic effort that did not reach Tier I by IVETF’s standards, and only 10.4% were both clinically diagnosed and retrospectively classified with epilepsy (12). Kearsley-Fleet et al. (1) identified that only 2% of epileptic cases underwent MRI screening within primary care settings, potentially due to inaccessibility of diagnostic resources in these settings.

For epilepsy protocols across referral populations, 60% of German dogs received a clinical diagnosis but severe epileptic cases, such as status epilepticus, were more likely to receive advanced diagnostic work (8). Hamamoto et al. (9)’s paper applied the IVETF tiers to retrospective data, finding that out of 172 dogs with IE: two dogs fulfilled Tier I, 13 dogs fulfilled Tier II, and four dogs fulfilled Tier III. Of those that fulfilled Tier II, all had insufficient blood tests for Tier I but still received MRI and CSF analyses (9). Thus, guidelines for IVETF IE diagnosis were not consistently followed, as fulfilling Tier I is a prerequisite for Tier II diagnostic confidence. However, in some cases, dogs underwent these advanced evaluations before being referred (9).

### 3.3 Socioeconomic status

Socioeconomic status impacted the quality of treatment received. In Erlen et al. (12)’s study. 74.0% (2,096/2,834) of canines had no record of insurance status, 18.4% (521/2,834) were insured, and 7.7% (217/2,834) were uninsured. Overall, 49.9% (1,415/2,834) of incident cases obtained an IVETF Tier I or higher evaluation, with 22.6% (320/1,415) of these canines being insured. Researchers identified that insured animals were 1.50 times more likely to receive a Tier I or higher diagnostic evaluation (12). Insurance status largely influences the clinical outcome of animals within these settings (1, 12). Having pet insurance mitigates financial constraints for owners and primary care clinics alike, allowing access to more advanced medical care (1, 12).

In referral populations, the owner’s socioeconomic status is at the forefront as often these specialized practices are more expensive due to the availability of more intensive evaluations (10). Owners of dogs susceptible to disorders are more likely to spend more on intensive treatment strategies than others (10). Conversely, owners of mixed-breed dogs or owners of lower socioeconomic status may have less incentive to continue with specialized care due to financial constraints or a lack of a recognized predisposition for their mixed-breed dog (10).

### 3.4 Cultural differences

Cultural differences were seen in breed preferences, approaches to treatment, and pet health insurance. Epileptic canines in Japan had a median lifespan of 13.0 years (9), in comparison to 7.6 and 7.0 years calculated in previous Denmark studies (6, 19). Longer lifespans and higher survival rates were suggested to be due to Japan’s considerable small breed population as well as the lack of euthanasia (4/100 euthanized), as euthanasia is negatively regarded in this culture (9).

In Sweden, veterinary medical insurance is common, with 40% of dogs insured by Agria, the main company (4). With different cultures comes differing popularity of breeds and their purpose (for work or companionship), factors that affected treatment outcome. Working breeds had a shorter life expectancy due to an increased likelihood of euthanasia while breeds kept as companions tended to receive epilepsy treatment and live longer (4). This distinction could be due to differences in owner requirements for their animal and their own socioeconomic status.

### 3.5 Regional differences

Regional differences were most prominent in areas of lower density and areas with fewer veterinary services. Heske et al. (4) found that where the dog resided, and their purpose, heavily influenced epilepsy evaluation. Their study analyzed rural and urban areas across three regions of Sweden (North rural, Central and South) to understand how geographical location impacted epilepsy evaluation (4). South and Central areas had much higher life (South: 750 cases rural, 218 cases urban; Central: 760 cases rural, 254 cases urban) and veterinary care (South: 1,555 cases rural, 713 cases urban; Central: 1,494 cases rural, 963 cases urban) insurance claims, while claims were less common in the North rural region (Life: 345 cases; Veterinary Care: 288 cases) (4). This region had fewer clinics and specialized services with larger distances between them (4). Due to this, epileptic dogs experienced a 22% (hazard ratio: 1.22) higher hazard of death within this location, affecting the rate of epilepsy diagnosis and prevalence calculations (4).

## 4 Discussion

This review found that there are few studies that have attempted to estimate the true general population of epileptic canines, that differences in cultural norms impact treatment decisions, that sociodemographic variables (such as location) impact accessibility to veterinary care, thereby affecting mortality and diagnostic rates, and lastly, that there has been inconsistency in the application of standards for epilepsy diagnosis across veterinary care settings.

### 4.1 Distribution of countries

Monitoring canine epilepsy prevalence in primary care settings is ongoing in the United Kingdom, as seen by the progression of studies through 2013, 2018, and 2020 (1, 11, 12). Only the Sweden study presented a mixed population perspective, providing a leading example in more generalizable epidemiological research on canine epilepsy prevalence (4). Conversely in mixed populations, referral animals are a subset of the primary care population. This provides an opportunity to investigate factors contributing to acceptance of referral and the spectrum of care that is accessed. Comparing these studies shows how little is known regarding the true population of epileptic dogs across countries. A future research focus on primary care and mixed populations will help to understand primary healthcare standards, referral factors, the true prevalence of canine epilepsy, and how it manifests globally.

### 4.2 Diagnostic evaluation and influences on epilepsy outcomes

Given the recency of the 2015 IVETF proposals, many earlier studies did not have a uniform standard for diagnosing and managing canine epilepsy. Due to the lack of standardized protocols, a large amount of variability in the levels of care is present across primary and referral clinics. This inconsistency affects both how many dogs receive sufficient assessment, and prevalence data collected from these clinics. IVETF guidelines can be hard to quickly consult due to their density rendering them largely inaccessible to primary care veterinarians causing a limited awareness of these protocols (20). Tier I diagnostics can be easily run within primary care environments, but Tier II and higher require more resources or are not available to primary care practices (20). Erlen et al. (12)’s findings that only 10.4% of incident cases were both clinically diagnosed and retrospectively classified with epilepsy reinforces the importance of improved veterinary education on IVETF protocols and access to resources within primary care settings. Regarding resources, educating owners about the benefits of pet insurance may reduce economic limitations that prevent access to key diagnostic procedures (21).

Cultural norms may impact epilepsy outcomes, via influence on decisions such as purchasing health insurance or breed preferences. Based on the animal’s purpose, a working dog may be less likely to be treated if their performance is affected, impacting the caregiver’s livelihood and finances (4). In Japan, euthanasia is negatively regarded, likely swaying the owner’s decision to pursue further treatment instead (9).

### 4.3 Is this information generalizable?

Although all population types are useful for different epidemiological questions, the primary care populations provided the most generalizable information regarding canine epilepsy and/or seizure prevalence due to an unselected patient population (4). In these populations, many epileptic cases can be overlooked as limited financial resources and inaccessibility of Tier I or higher diagnostic certainty impacts their diagnostic precision (12, 22).

Referral populations are specialized settings that are more likely to treat severe epileptic cases. These environments select dogs with generally more motivated or well-resourced owners, which reduces the generalizability of data collected (11). Additionally, while the mixed population study (3) included a mix of primary and referral patients, this population comprised dogs insured by a sole insurance company, thereby limiting its generalizability.

## 5 Limitations

### 5.1 Individual papers

All included studies were retrospective chart reviews, except for one cohort study (4). Notably, only five out of seven included articles (4, 8, 9, 11, 12) gave a total population prevalence of epilepsy and/or seizures encompassing all etiologies. Of these five articles, the estimates from the primary care (0.62 and 0.82%) and mixed population (0.75%) papers were of similar values (4, 8, 9), while the estimates from referral populations (1.9 and 2.6%) showed a higher population prevalence as expected (11, 12). Kearsley-Fleet et al. (1) and Bellumori et al. (10) only provided the prevalence of IE (0.62 and 1.04% respectively), following a similar population prevalence trend as above.

Vet Compass records were used in all primary care articles. Insurance claims were used in the Heske et al. (4)’s study. Notably, these records are originally not intended for research use, therefore there may be important data gaps between what is recalled by the caregiver, recorded by the veterinarian, and information needed for producing robust effect estimates through appropriate control of potential confounding variables (11). Given that the identification and nature of confounding variables is dependent on the exposure and outcome being studied, it is unlikely that medical records will contain all necessary information for confound control, leaving some level of unmeasured and uncontrolled confounding and ultimately biased estimates. Additionally, the results from these papers could underestimate the true absolute period prevalence of seizure/epilepsy occurrence (11). As dogs cannot self-report and recorded occurrence is reliant on owner description, seizure events can go unrecognized or be falsely classified (3, 4, 11). Alternatively, gaps within insurance claims are plausible, as cases not recorded in the database may have had seizures, but the cost of care may have not exceeded the deductible, meaning care was not reimbursed, potentially causing epilepsy prevalence to be underestimated in this database (23).

Three of the included papers (1, 4, 8) were run before the IVETF protocols were proposed, and in one paper (9), the IVETF guidelines were inconsistently applied. Therefore, the standard of diagnostic care is limited or inconsistent. Prospective cohort or case–control studies could systematically apply IVETF standards as cases occurred.

Additionally, four of the included papers (4, 8–10) had a restricted population scope, limiting the generalizability of their results. This was seen through the focusing on specific etiologies of canine epilepsy/seizures (i.e., status epilepticus, idiopathic epilepsy, or exclusion of reactive seizures) or canine populations (i.e., the United States west coast dog population or dogs insured by Agria).

### 5.2 Our review

The language limitation of this review means that reports may have been missed on canine epilepsy prevalence in primary care or referral populations. Additionally, the design and implementation of the search strategy may not have been exhaustive enough, leading to missed articles. Mitigation of this limitation was through hand-searching reference lists of relevant articles. All studies evaluated focused on retrospective analyses of veterinary medical records or insurance claims. Solely reliant on retrospective data, these populations are not able to capture all cases in the general population, providing a major limitation to the studies and, by association, this review.

## 6 Conclusion

More epidemiological research on primary care and mixed populations is needed to establish robust estimates of canine epilepsy prevalence in dog populations. In addition, more veterinary education is needed to standardize the IVETF epilepsy protocols’ use in primary care settings alongside enhanced owner education on pet insurance to help reduce economic barriers to epilepsy diagnosis and treatment. Importantly, socioeconomic status, geographical location and culture can have a large impact on the quality of life of a dog living with epilepsy.
